# Comparison of the Latarjet Procedure and Iliac Crest Bone Graft Transfer in Bone Bankart Lesions in Recurrent Anterior Shoulder Dislocations

**DOI:** 10.7759/cureus.66176

**Published:** 2024-08-05

**Authors:** Mahmut Tuncez, Ömer Berkay Bayraktar

**Affiliations:** 1 Department of Orthopedics and Traumatology, Izmir Katip Celebi University Ataturk Training and Research Hospital, Izmir, TUR

**Keywords:** bone transfer, latarjet, bony bankart, bankart, shoulder dislocation

## Abstract

Objective: There are various treatment modalities for bony Bankart lesions following glenohumeral joint dislocations. In this research, we aimed to compare the radiological and clinical results of patients who underwent the Latarjet procedure and iliac crest bone graft transfer.

Materials and methods: Clinical and radiological data of 15 patients were retrospectively evaluated. Inclusion criteria were a history of at least two dislocations, being between 18-65 years of age and having at least 10% glenoid bone loss. The exclusion criteria were defined as follows: patients who underwent only soft tissue procedures, patients who did not attend the follow-up, patients with other pathology in the upper extremity (fracture, cuff tear, etc.), and patients with a follow-up period of less than 12 months time. Nine patients underwent the Latarjet procedure and six underwent iliac crest bone graft transfer. Clinical evaluation, age, gender, body mass index, range of motion, Quick Disabilities of the Arm, Shoulder and Hand (QDASH) score, Constant score, and Visual Analogue Scale (VAS) score were evaluated. Radiographic evaluation was performed with preoperative and postoperative direct radiographs and computed tomography. Mann-Whitney U test, t-test, and Fisher exact tests were used for group comparisons.

Results: The mean age of the patients was 32.6 years and the mean follow-up period was 24.9 months. When the two groups were compared, no statistical difference was found in terms of age, gender, body mass index, range of motion, Constant score, VAS score, glenoid cartilage stepping, and bone defect (p>0.05). The operation time was longer in the iliac crest bone graft transfer group compared to the Latarjet group (p<0.05).

Conclusion: Latarjet and iliac crest bone graft transfer can be used successfully in the treatment of bony Bankart in recurrent anterior shoulder dislocations. The operation time is longer in iliac crest bone graft transfer compared to the Latarjet procedure.

## Introduction

Nowadays, in the emergency department, half of the patients presenting with dislocations are shoulder dislocation patients. In 90% of shoulder dislocations, anterior shoulder dislocation occurs and is mostly treated with closed reduction. The pathology causing the dislocation is injury of the anterior glenoid labrum or Bankart lesion. However, an associated bone defect in the glenoid and/or humeral head may occur [1]. Soft tissue stabilisation procedures for the treatment of anterior shoulder instability have proven to be less effective in patients with extensive glenoid bone loss [2]. Therefore, in case of bone loss, bone tissue procedures are needed instead of soft tissue procedures. These procedures are of two types: coracoid transfer techniques and free bone grafting techniques. The commonly used coracoid transfer procedure is the modern type Latarjet procedure, which involves transferring the coracoid to the anterior glenoid rim through a permanent horizontal incision of the subscapularis muscle [3,4]. Other methods include the modern Eden-Hybinette procedure, a commonly used free bone transfer technique, or the iliac crest bone graft transfer (ICBGT), which involves harvesting an autologous bone graft from the iliac crest and transferring it to the anterior glenoid margin [5,6]. The aim of Latarjet and ICBGT is to create a mechanical block on the anterior glenoid margin. The aim of this research was to compare clinically and radiologically recurrent shoulder dislocation patients treated with these two techniques.

## Materials and methods

Our research was conducted as a retrospective controlled study with approval from Izmir Katip Celebi Universıty Non-Interventional Clinical Studies Institutional Review Board (approval number 26). Of the 35 patients operated on for anterior shoulder dislocation in our clinic between January 2021 and May 2023, 15 patients operated on for bone Bankart lesions were included in the study. Inclusion criteria were a history of at least two dislocations, being between 18-65 years of age, and having at least 10% glenoid bone loss. Exclusion criteria were as follows: patients treated with only soft tissue procedure, patients who did not attend the follow-up, patients with other pathology in the upper extremity (old fracture, cuff tear, neurological deficit), and patients with a follow-up period of less than 12 months.

**Figure 1:**
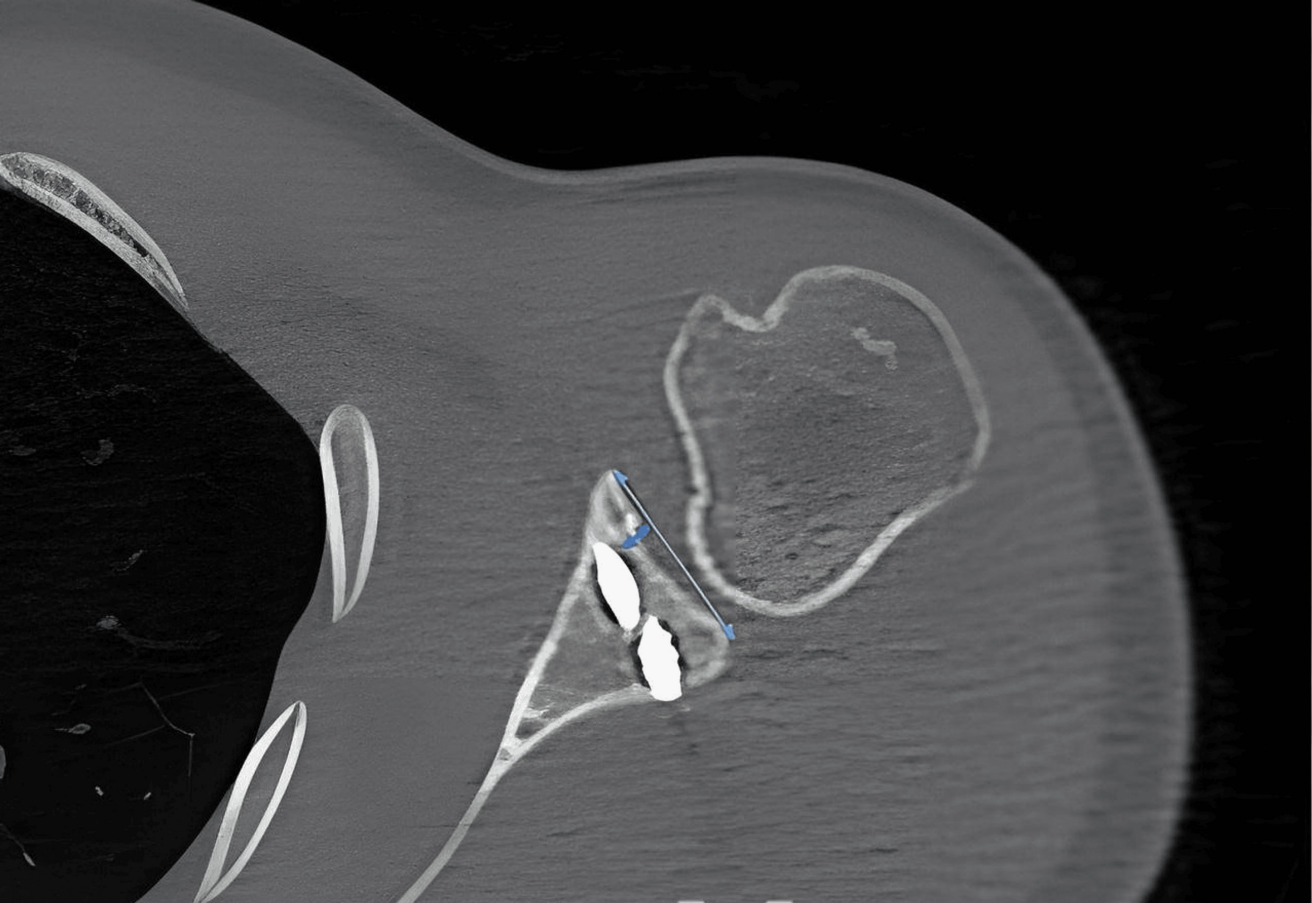
The mediolateral distance between the graft surface and the bony glenoid surface: glenoid cartilage stepping

Nine patients were treated with the Latarjet procedure and six with ICBGT. Clinical evaluation was performed by age, gender, body mass index, range of motion, Quick Disabilities of the Arm, Shoulder and Hand (QDASH) score, Visual Analogue Scale (VAS) score and Constant score. Radiographic evaluation included preoperative and postoperative direct radiographs and computed tomography analysis. On computed tomography; bone defect percentage and postoperative glenoid cartilage stepping were measured. The glenoid bone defect was measured using the Pico method [7]. Glenoid cartilage stepping was measured between the intact glenoid and the graft using transverse computed tomography sections (Figure 1).

Mann-Whitney U test, t-test, and Fisher exact test were used for group comparisons. In all reported tests, p<0.05 was considered statistically significant.

**Figure 2:**
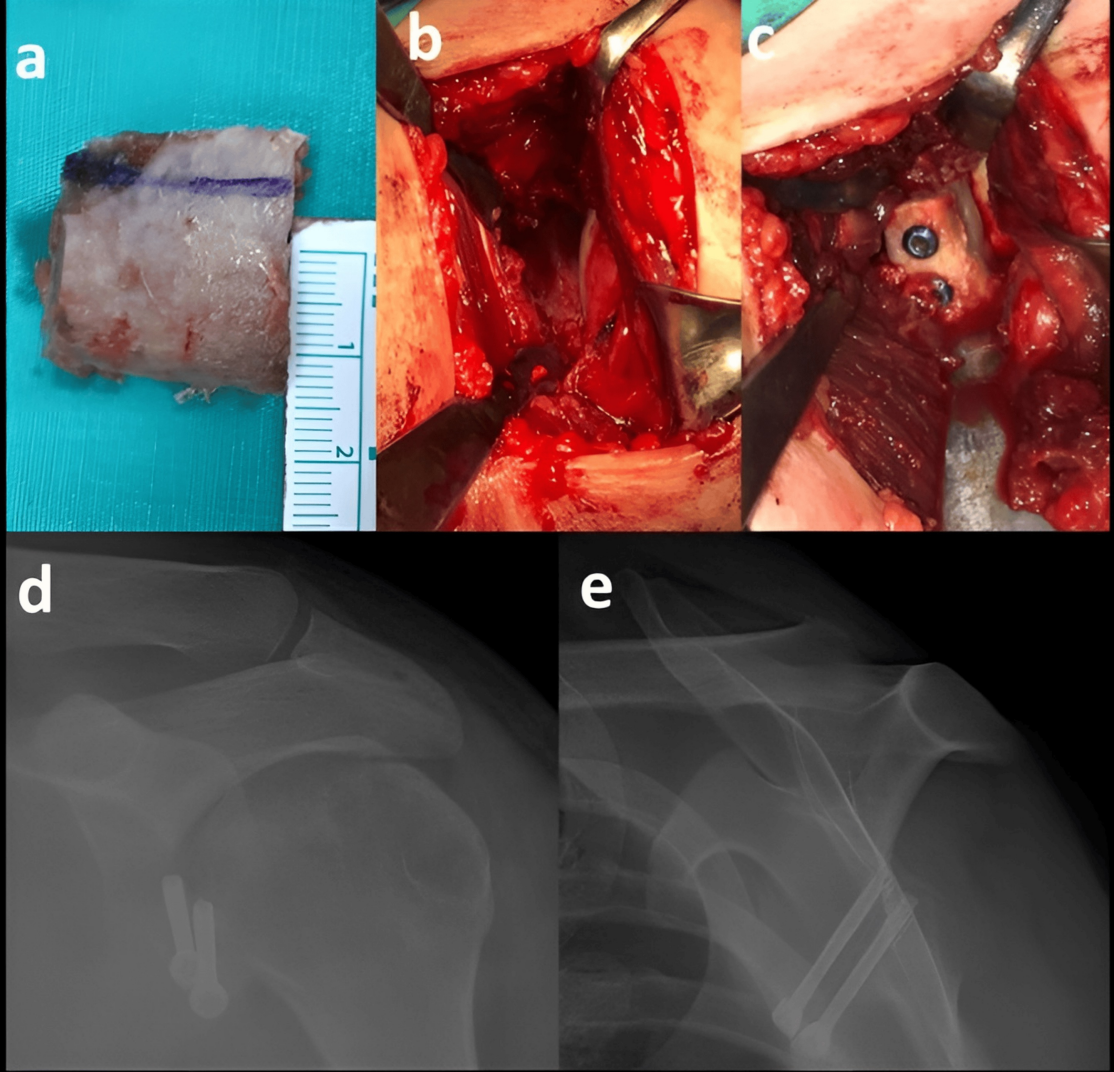
Intraoperative images and postoperative radiographs a) Autogenous bone graft (20 x 15 mm) taken from the iliac crest. b) Preparation of the anterior glenoid rim. c) Fixation of the autogenous bone graft with two 3.5 mm cannulated screws. d-e) Postoperative direct radiographs.

The Latarjet procedure was performed with a 6-8 cm incision between the coracoid and the anterior axillary line, leaving the pectoralis minor tendon-free. Conjoint tendon and coracoacromial ligaments were left intact and osteotomy of the coracoid near the base was performed. The glenohumeral joint was reached after vertical separation of the capsule with the transverse insertion of the subscapularis muscle. Then, the fibrous tissues in the anteroinferior part of the glenoid were removed and the blood supply of the glenoid bone with the burr was ensured. Following the transposition of the coracoid to the anterior glenoid margin flush with the glenoid articular surface and fixation to the glenoid with two screws, the procedure was terminated with anatomical closure of the subscapularis muscle. In the ICBGT technique, a rectangular (20x15 mm) tricortical bone graft was taken from the iliac crest, and the bone block was fixed to the anterior glenoid anteriorly in the same manner as the latarjet procedure (Figure 2).

Patients were followed up with passive shoulder movements between two and six weeks after two weeks in a shoulder arm sling. In the sixth week, active strengthening exercises were started.

## Results

The mean age was 32.6 ± 8.5 years and the mean follow-up period was 24.9 ± 10.2 months. The mean Constant score was 84.2 ± 10.4, mean QDASH 15.1 ± 14.2, mean VAS 2.8 ± 1.7, mean internal rotation degree 42.3 ± 12.7 and mean glenoid bone defect percentage 15.2 ± 2.9%. Other categorical and demographic changes are shown in Table 1. These are the final follow-up results of all patients in both patient groups.

**Table 1:** Demographic and categorical data of all patients QDASH: Quick Disabilities of the Arm, Shoulder and Hand, VAS: Visual Analogue Scale, SD: Standard Deviation, Min: Minimum, Max: Maximum, IQR: Inter Quantile Range

Parameters	Mean	Median	SD	Min	Max	IQR
Age (years)	32.6	32	8.5	21	47	26-39
Body mass index (kilogram/meter^2^)	24.01	24.4	4.8	16.7	31.4	19-27
Duration of surgery (minute)	59.6	60	11.7	40	75	50-70
Duration of hospital stay (day)	2.9	3.0	0.79	2	4	2-4
Total follow-up time (month)	24.9	27	10.2	12	42	13-34
Number of instability episodes	5.06	5	2.4	2	11	3-7
Glenoid cartilage Stepping (mm)	0.06	0	0.16	-0.2	0.3	0-0.2
Glenoid Bone defect (%)	15.2	15	2.9	10	22	13-17
Constant score	84.2	84	10.4	65	100	79-94
QDASH	15.1	6.8	14.2	2.3	36.4	4.5-36
VAS	2.8	3	1.7	1	8	1-3
Forward elevation (degree)	168	180	18.5	110	180	160-180
Abduction (degree)	154	160	15.9	100	170	150-160
Internal rotation (degree)	42.3	35	12.7	30	60	30-55
External Rotation (degree)	76.6	80	4.8	70	80	70-80
Back to work (month)	2.3	2	0.7	0.5	3.0	2-3

When the patients were analyzed as latarjet and ICBGT, no statistical difference was found in terms of age, gender, body mass index, range of motion, Constant score, VAS score, glenoid cartilage stepping, and bone defect (p>0.05) (Table 2). However, we found that the ICBGT group had a statistically longer operation time than the latarjet group (p<0.05) (Table 2). No postoperative complications were observed in either patient group (wound problem, infection, arthritis, neurological disorder, dislocation, etc.).

**Table 2:** Comparison of group characteristics ICBGT: Iliac Crest Bone Graft Transfer, QDASH: Quick Disabilities of the Arm, Shoulder and Hand, VAS: Visual Analogue Scale.

Parameters	Latarjet (n=9)	ICBGT (n=6)	p
Age (years)	34±8.4	30.6±9.1	0.483
Body mass index (kilogram/meter^2^)	23,9±5,3	24,1±4,4	0.944
Duration of surgery (minute)	54.4±6.8	67.5±13.6	0.028
Duration of hospital stay (day)	3±0,8	2,8±0,7	0.707
Total follow-up time (month)	23,1±12	27,6±6,9	0.419
Number of instability episodes	5,3±2,6	4,6±2,1	0.617
Glenoid cartilage Stepping (mm)	0±0,1	0±0,1	0.847
Bone defect (%)	15,3±3,6	15,1±1,7	0.918
Constant score	82,4±11,3	87±9,2	0.430
QDASH	11,3±11,6	20,8±17	0.301
VAS	2,2±1	3,6±2,3	0.157
Forward elevation (degree)	168,8±23,1	166,6±10,3	0.240
Abduction (degree)	150±19	160±6,3	0.158
Internal rotation (degree)	44,4±13	39,1±12,8	0.461
External Rotation (degree)	76,6±5	76,6±5,1	>0.999
Back to work (month)	2,4±0,5	2±0,9	0.465

## Discussion

The main finding of our study was that there was no difference between Latarjet and ICBGT clinically and radiologically. However, it was found that the operation time was statistically significantly prolonged in the ICBGT group compared to the Latarjet group. We attribute this difference to the prolonged operation time of the ICBGT group because two different incisions were used during the operation. In a previous prospective study by Moroder et al., the same two techniques were compared, and similar results were obtained with our study and it was shown that there was no difference in clinical outcome [8]. Moroder et al. obtained better results in shoulder internal rotation in the ICBGT group compared to the Latarjet group and attributed this to the permanent division of the subscapularis tendon in the latarjet procedure [8]. In our study, we did not find any statistical difference between the range of motion and internal rotation between both groups.

The two surgical techniques were performed by one senior surgeon with a special focus on shoulder surgery in our hospital. The duration of surgery was longer in the ICBGT group than in the latarjet group. We think that the reason for this is that the bone graft was taken from the iliac crest through a separate incision. In the literature, we could not find any study comparing the two groups in terms of surgical times. However, Razaenian et al. compared latarjet and arthroscopic ICBGT and found that arthroscopic ICBGT had a longer duration of surgery [9].

The selected cut-off value of 10% glenoid bone loss is not commonly accepted. A defect size of 13.5% has been referred to as "subcritical bone loss" by some authors [10,11]. These results show that when only soft tissue stabilization is performed, significantly worse results occur in patients with high activity in the presence of bone Bankart [10,11]. The mean age of our patient group was 32.6 years and mostly male patients with high activity. For these reasons, the authors used this cut-off value. When the two groups were evaluated in terms of postoperative instability, no postoperative subluxation was observed in any patient. We think that this is due to the fact that both techniques were successful in terms of stabilization [8,12-15].

The limitations of the study include the relatively small number of patients and its retrospective nature. However, we think that our study may contribute to the literature since these surgeries for bone defects in anterior shoulder dislocation are rarely performed routinely. In the future, prospective studies with a larger number of patients will be guiding to evaluate the effectiveness of both techniques in bone Bankart lesions.

## Conclusions

There is still no gold-standard treatment for anterior shoulder instability with glenoid bone loss. Latarjet and ICBGT procedures do not show any difference in clinical and radiological outcomes except for a significantly longer operative time in the ICBGT group. Both procedures can be performed successfully in bone Bankart lesions.

## References

[1] Provencher MT, Bhatia S, Ghodadra NS (2010). Recurrent shoulder instability: current concepts for evaluation and management of glenoid bone loss. J Bone Joint Surg Am.

[2] Burkhart SS, De Beer JF (2000). Traumatic glenohumeral bone defects and their relationship to failure of arthroscopic Bankart repairs: significance of the inverted-pear glenoid and the humeral engaging Hill-Sachs lesion. Arthroscopy.

[3] Latarjet M (1954). A propos du traitement des luxations récidivantes de l’épaule. Lyon Chir.

[4] Young AA, Maia R, Berhouet J, Walch G (2011). Open Latarjet procedure for management of bone loss in anterior instability of the glenohumeral joint. J Shoulder Elbow Surg.

[5] Weng PW, Shen HC, Lee HH, Wu SS, Lee CH (2009). Open reconstruction of large bony glenoid erosion with allogeneic bone graft for recurrent anterior shoulder dislocation. Am J Sports Med.

[6] Auffarth A, Schauer J, Matis N, Kofler B, Hitzl W, Resch H (2008). The J-bone graft for anatomical glenoid reconstruction in recurrent posttraumatic anterior shoulder dislocation. Am J Sports Med.

[7] Magarelli N, Milano G, Sergio P, Santagada DA, Fabbriciani C, Bonomo L (2009). Intra-observer and interobserver reliability of the 'Pico' computed tomography method for quantification of glenoid bone defect in anterior shoulder instability. Skeletal Radiol.

[8] Moroder P, Schulz E, Wierer G, Auffarth A, Habermeyer P, Resch H, Tauber M (2019). Neer Award 2019: Latarjet procedure vs. iliac crest bone graft transfer for treatment of anterior shoulder instability with glenoid bone loss: a prospective randomized trial. J Shoulder Elbow Surg.

[9] Razaeian S, Tegtmeier K, Zhang D, Bartsch S, Kalbe P, Krettek C, Hawi N (2022). Open latarjet procedure versus all-arthroscopic autologous tricortical iliac crest bone grafting for anterior-inferior glenohumeral instability with glenoid bone loss. J Orthop Surg (Hong Kong).

[10] Shaha JS, Cook JB, Song DJ, Rowles DJ, Bottoni CR, Shaha SH, Tokish JM (2015). Redefining "critical" bone loss in shoulder instability: functional outcomes worsen with "subcritical" bone loss. Am J Sports Med.

[11] Dickens JF, Owens BD, Cameron KL, DeBerardino TM, Masini BD, Peck KY, Svoboda SJ (2017). The effect of subcritical bone loss and exposure on recurrent instability after arthroscopic Bankart repair in intercollegiate American football. Am J Sports Med.

[12] Degen RM, Camp CL, Werner BC, Dines DM, Dines JS (2016). Trends in bone-block augmentation among recently trained orthopaedic surgeons treating anterior shoulder instability. J Bone Joint Surg Am.

[13] Nzeako O, Bakti N, Bawale R, Singh B (2019). Bone block procedures for glenohumeral joint instability. J Clin Orthop Trauma.

[14] Ramhamadany E, Modi CS (2016). Current concepts in the management of recurrent anterior gleno-humeral joint instability with bone loss. World J Orthop.

[15] Moroder P, Blocher M, Auffarth A, Hoffelner T, Hitzl W, Tauber M, Resch H (2014). Clinical and computed tomography-radiologic outcome after bony glenoid augmentation in recurrent anterior shoulder instability without significant glenoid bone loss. J Shoulder Elbow Surg.

